# Comorbidities in multiple sclerosis: acquired hepatocerebral degeneration mimicking disease progression

**DOI:** 10.1007/s10072-026-09284-x

**Published:** 2026-07-31

**Authors:** Maximilian Einsiedler, Alexandros Polymeris, Nikolaos Raptis, Anika Beyer, Denise Becker, Lisette Krassenburg, Isabelle Panne, Mustafa Ahmed Mahmutoglu, Johanna Maria Lieb, Stephan Rüegg, Tobias Derfuss, Jens Kuhle

**Affiliations:** 1 Multiple Sclerosis Centre and Research Center for Clinical Neuroimmunology and Neuroscience (RC2NB), Neurology, Departments of Biomedicine and Clinical Research, University Hospital and University of Basel, Petersgraben 4, Basel, CH-4031 Switzerland; 2 Department of Gastroenterology and Hepatology, University Hospital Basel, Basel, Switzerland; 3 Division of Diagnostic and Interventional Neuroradiology, Clinic for Radiology and Nuclear Medicine, University Hospital Basel, Basel, Switzerland

**Keywords:** Progression independent of relapse activity, Neurofilament light chain, Glial fibrillary acidic protein, Hepatocerebral degeneration, Hepatic encephalopathy

## Abstract

A 63-year-old patient with relapsing-remitting multiple sclerosis (MS) and comorbid liver cirrhosis presented with progressive cognitive decline, gait disturbance and elevated serum neurofilament light chain (NfL), suggesting disease progression independent of relapse activity (PIRA). Development of acute hepatic encephalopathy led to reconsideration of the cause of previous clinical worsening and MRI revealed progressive basal ganglia manganese accumulation, establishing the diagnosis of acquired hepatocerebral degeneration, retrospectively challenging the PIRA diagnosis. Careful evaluation of comorbidities is warranted in older MS patients, as progressive worsening and high NfL may indicate alternative etiologies, particularly if GFAP (glial fibrillary acidic protein) stays within normal range.

We present a 63-year-old male patient diagnosed at age 57 with relapsing-remitting multiple sclerosis (MS) according to the McDonald 2017 criteria [1]. He had a 10-year history of recurrent paresthesia of the arms, severe fatigue, and bladder-symptoms, before presenting with a subacute myelitis, upper-extremity sensory deficits and positive Lhermitte sign, leading to work-up showing multiple demyelinating lesions on cerebral and spinal MRI. Lumbar puncture showed CSF-specific oligoclonal bands. Visual evoked potentials showed delayed P100 latencies. Medical history included obesity, dyslipidemia, hypertension and a maternal MS diagnosis.

The patient was started on dimethyl fumarate. Although yearly imaging showed no evidence of new MS-associated lesions, he reported over the following five years a gradual worsening of gait, bladder-symptoms and cognitive abilities. Neurological examination showed gait- and limb-ataxia and intention tremor. Walking distance declined from 5 km to 500 m. Expanded Disability Status Scale (EDSS) score increased from 3.0 to 4.0. Serum neurofilament light chain (NfL) levels were within normal range [2] three years after the diagnosis (7.9 pg/mL, Z score − 1.23, 11th percentile), then increased within 1.5 years to the 99.1st percentile (27.9 pg/ml, Z score 2.37, Fig. 1), mirroring the clinical worsening. Cognitive function worsened, the Montreal Cognitive Assessment (MoCA) decreasing from 27/30 to 18/30. Together, this led to diagnosis of progression independent of relapse activity (PIRA) and treatment was escalated to ocrelizumab. In parallel, a diagnosis of metabolic dysfunction-associated steatohepatitis (MASH) and associated liver cirrhosis was made after abdominal imaging performed for an unrelated indication showed extensive portosystemic collateral circulation. Due to the apparent benign course (Child-Pugh A), no specific treatments were introduced and ammonia was not measured.

**Fig. 1:**
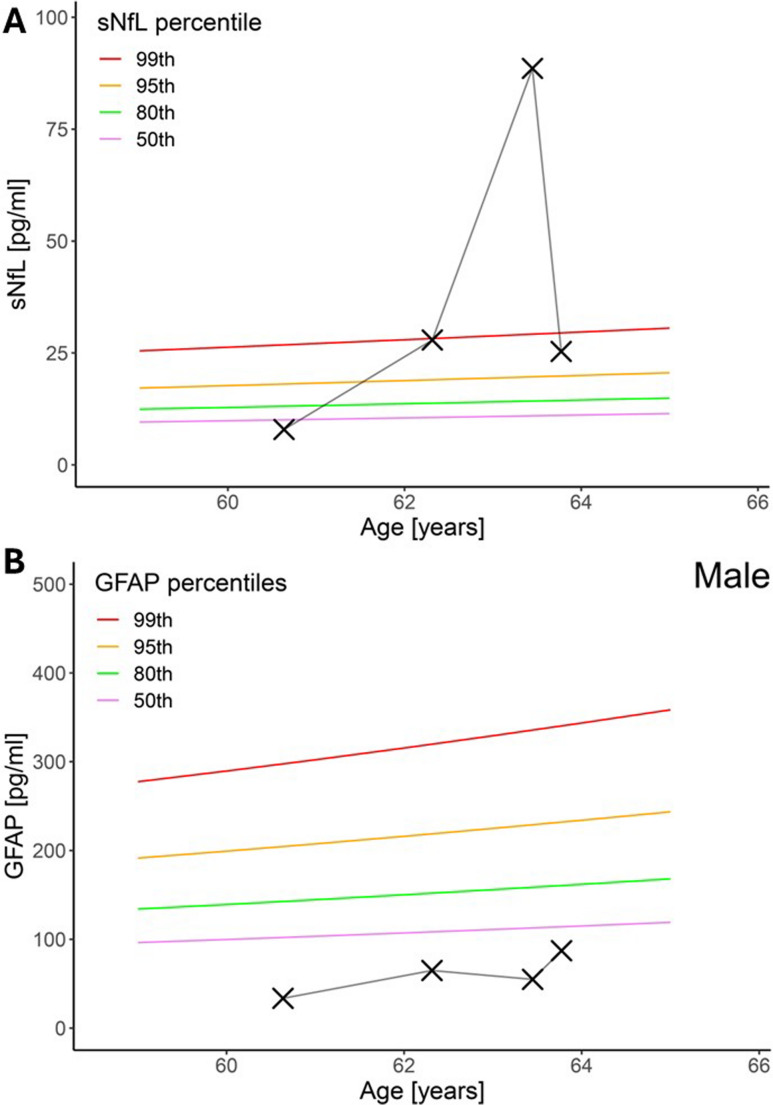
Evolution over time of serum neurofilament light chain and serum glial fibrillary acidic protein. Legend: **(A)** Serum NfL levels over time. Colored lines represent values of healthy controls [2] (red: 99th percentile, yellow: 95th percentile, green: 80th percentile, purple: 50th percentile). Serum NfL increased substantially over time, mirroring progressive clinical worsening. The peak corresponded to the episode of overt hepatic encephalopathy. **(B)** Serum GFAP levels over time. Colored lines represent values of healthy controls [3] (red: 99th percentile, yellow: 95th percentile, green: 80th percentile, purple: 50th percentile). Serum GFAP was within normal ranges at any timepoint. Abbreviations: GFAP: glial fibrillary acidic protein. sNfL: serum neurofilament light chain

Following the first ocrelizumab course (2*300 mg intravenously), severe lymphopenia prompted treatment suspension. 12 months later, further worsening of cognition and gait occurred (walking distance 280 m), leading to an EDSS of 5.0. Neurological examinations showed marked limb and gait ataxia, dysexecutive syndrome, dysarthria, dysdiadochokinesia, bradykinesia and intention tremor. NfL further rose to the 99.96th percentile (88.6 pg/ml, Z score 3.35, Fig. 1). MoCA score was 16/30. Neuroimaging indicated no new MS disease activity.

During this worsening, laboratory testing showed hyperammonemia (201 µmol/l), elevated bilirubin (36.5 µmol/l) and international normalized ratio (INR; 1.7), low albumin (27 g/l). Liver enzymes were only slightly elevated (aspartate aminotransferase at 65 U/I), without significant changes after ocrelizumab. EEG showed clear abnormalities with epileptiform discharges, occasionally progressing to brief subclinical seizures. Lactulose and rifaximin were started for acute hepatic encephalopathy (HE); levetiracetam for seizure management. Cirrhosis was classified as Child-Pugh C11.

Retrospective review of brain MRIs over the previous 5 years showed progressively increasing basal ganglia T1-hyperintensity consistent with probable manganese deposits (Fig. 2), correlating with progressive clinical worsening and leading to the diagnosis of acquired hepatocerebral degeneration (AHD). After treatment of the acute episode, laboratory testing, gait and cognition partially improved. Notably, NfL declined substantially, but remained high compared to healthy controls (25.3 pg/ml, 98.2nd percentile, Z score 2.10, Fig. 1). In contrast to NfL, and during the whole period, serum glial fibrillary acidic protein (GFAP) was within normal range [3] (last follow-up: Z score − 0.52, 30th percentile, Fig. 1).

**Fig. 2:**
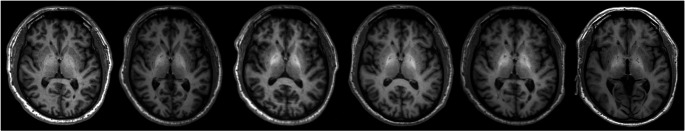
Brain MRI evolution over time. Legend: Axial T1-weighted brain MRI scans taken at MS diagnosis and at 18, 30, 42, 54, and 64 months (left to right). The images show progressively increasing hyperintensities in the globus pallidus, consistent with probable manganese deposition associated with acquired hepatocerebral degeneration. Abbreviations: MRI: magnetic resonance imaging. MS: multiple sclerosis

## Discussion

We report a patient with MS, developing progressive neurological symptoms attributable to concomitant AHD.

AHD is a rare but under-diagnosed entity complicating chronic liver disease [4]. Typical MRI-abnormalities include high signal intensity in the globus pallidum on T1-weighted images, likely as a reflection of increased manganese concentration and altered glutamine/glutamate, myo-inositol and choline concentrations [5]. A portosystemic shunt circulation is closely linked to these deposits and often required for the disease to develop [4–6], even with minimally affected liver function [6]. Clinical symptoms are varied, parkinsonism, ataxia, cognitive impairment and dysexecutive syndrome being the main features [4–6]. In our case, the patient had a slow worsening of gait, cognition and executive functions, retrospectively attributable to AHD.

Intermittent overt HE is often associated [6]. During HE episodes, neurological impairment is usually much more severe, paraclinical findings including hyperammonemia and pathological EEG confirm the diagnosis. In our case, this acute episode with dysarthria, markedly worsened ataxia and severe cognitive fluctuations, prompted reconsideration of the progressive course initially attributed to MS.

NfL is a marker of neuro-axonal damage and increasingly used in MS to assess the risk of disease activity and the response to disease-modifying treatment. NfL is also related to disease progression, notably PIRA [7]. However, NfL is not specific to MS; several neurodegenerative disorders also lead to substantial elevations. NfL has been recently described as a marker of occurrence of HE in patients with liver cirrhosis and as a potential indicator of response to treatment with rifaximin [8, 9]. It is likely that NfL is also capturing more chronic neurodegenerative processes occurring in AHD. This is underlined by the substantial NfL elevation observed in our patient even before the episode of overt HE. Interestingly, GFAP, a marker of astrocytic damage and activation, which has been repeatedly described as a relevant biomarker in the context of MS progression and PIRA [3, 7], was within normal ranges in our patient [3]. This could indicate differing pathophysiological mechanisms, and in our case further substantiates the possibility of clinical worsening being unrelated to MS progression.

MS diagnosis above age 60 is uncommon, but increasingly frequent, partly due to more precise diagnosis and improved treatments. In this population, PIRA is more frequently observed, specific treatments are less clearly defined and treatment-associated adverse events are not uncommon [10]. Correctly assessing MS diagnosis and MS progression is therefore of utmost importance. This population is however prone to several comorbidities that can interfere with the course of MS [10]. In patients with liver cirrhosis, the possibility of AHD explaining progressive neurological worsening should be considered in typical presentations. NfL, though very valuable in assessing treatment-efficacy in MS, should be interpreted with caution. Particularly, extremely high values without concomitant radiological activity and associated with normal GFAP could potentially indicate alternative etiologies, as was the case in our patient, and warrant further diagnostic work-up.

Our case underlines the importance of precisely assessing comorbidities in patients with MS, especially over the age of 60, to improve diagnostic accuracy of disease progression.

## Data Availability

Data associated with this manuscript is available from the corresponding author upon reasonable request.
